# Experiences of individuals with serious mental disorders in regular employment through the Individual Placement and Support model

**DOI:** 10.3389/fpsyt.2024.1423742

**Published:** 2024-09-10

**Authors:** María Jesús Melián Cartaya, Ángeles Arias Rodríguez, Armando Rodríguez Pérez, María Sánchez Suárez, Natalia Rodríguez-Novo, Yurena Rodríguez-Novo, Francisco Rodríguez Pulido

**Affiliations:** ^1^ Individual Placement and Support (IPS) Team, Cabildo of Tenerife, Santa Cruz de Tenerife, Spain; ^2^ Area of Preventive Medicine and Public Health, University of La Laguna, Santa Cruz de Tenerife, Spain; ^3^ Department of Cognitive, Social and Organizational Psychology, Faculty of Psychology, University of La Laguna, Santa Cruz de Tenerife, Spain; ^4^ Department of Nursing, Universidad de La Laguna, San Cristóbal de La Laguna, Spain; ^5^ Presidency of the Official College of Nursing of Santa Cruz de Tenerife, Santa Cruz de Tenerife, Spain; ^6^ Universitary Hospital of Nuestra Señora de la Candelaria, Santa Cruz de Tenerife, Spain; ^7^ Department of Internal Medicine, Dermatology and Psychiatry, University of La Laguna, Santa Cruz de Tenerife, Spain

**Keywords:** serious mental disorder, narratives, job satisfaction, qualitative research, combination of methodological strategies

## Abstract

**Introduction:**

This study shows the perspective, meaning and satisfaction perceived by people with Serious Mental Disorders during their experiences in regular employment.

**Methods:**

A mixed qualitative-quantitative methodology was used, applying semi-structured interview as qualitative information collection tool and the Indiana Job Satisfaction Scale as quantitative tool. The study period was from January 2021 to December 2022. A purposive sampling was performed with a sample of 24 people with Serious Mental Disorders who had obtained a job through an Individual Placement and Support (IPS) program during the study period. Semi-structured interview and the Indiana Job Satisfaction Scale were applied to this Serious Mental Disorder workers’ sample. The Indiana Job Satisfaction Scale was also applied to a group of 24 workers without mental disorders in the same working conditions who served as control group.

**Results:**

The results of the analyses of the Serious Mental Disorder workers’ narratives show that perception of work experience is conditioned by individual, environmental and social predictors, as well as external factors as determining variables. Quantitative results obtained by the Indiana Job Satisfaction Scale reveal levels of job satisfaction resembling those of the rest of workers without Serious Mental Disorder.

**Discussion:**

These findings reinforce the significance of employment in the recovery process for individuals with Serious Mental Disorders and emphasize the importance of understanding the subjective meaning individuals attribute to their work experiences.

## 1 Introduction

Since the term Serious Mental Disorders (SMD) was coined in the late 1970s, a wide variety of definitions have emerged, primarily focusing on three dimensions: diagnosis, disability, and duration. Goldman and colleagues were among the first to use this term, defining individuals with chronic mental disorders as “those suffering from severe and persistent mental illnesses that interfere with their daily living capabilities, such as self-care, interpersonal relationships, work, or education; and often require prolonged hospital or psychiatric treatments” (1). Subsequently, the National Institute of Mental Health (NIMH) (2) presented the most widely accepted definition, which has garnered the most consensus. This definition incorporates the three dimensions mentioned above:

- Diagnosis, referring to psychotic disorders (excluding organic ones) and certain personality disorders.
- Duration of illness and treatment, considering a period exceeding two years.
- Presence of disability, which pertains to moderate to severe impairment in work, social, and family functioning.

Nevertheless, no solid and uniform criteria have been established, and there is no international agreement on the definition of SMD.

From a clinical point of view, Serious Mental Disorders refer to three main groups of diagnoses: schizophrenia and psychotic disorders, bipolar disorder and major affective disorders, and other disorders such as personality disorders. However, the definition of Serious Mental Disorders cannot be based solely on a clinical approach, since it should also consider criteria such as the duration and extent of impact on employment, as well as social and family functioning. Therefore, interventions with people with SMDs should not focus exclusively on reducing symptoms, but also on increasing their wellbeing and promoting their integration in the community (3).

Employment is a key element in the recovery of people with serious mental disorders (4), as well as a means of integration and active participation in society. So, it is accepted that unemployment in people with serious mental disorders (SMDs) paves the way to social exclusion (5).

Employment plays several roles in the lives of people with SMDs: It involves an activity, provides structure, an adult role, promotes recovery, has a positive impact on social functioning and the degree of life satisfaction, and personal autonomy. It also facilitates community integration, promotes self-esteem, sense of belonging and social relations, increases income, and improves the quality of life. (6, 7). All in all, employment boosts social inclusion of people with SMDs who consider it a central element of social connection for survival and mental health recovery (8).

Sheltered employment is an alternative to open (competitive) employment, which is the usual form of employment. Competitive employment involves integrating people with disabilities into the regular labor market, where integration is fully realized through working alongside non-disabled colleagues on equal terms and under similar working conditions (9). Thus, most people with SMDs wish to have a competitive job (10, 11). This daily interaction with others under normal circumstances has a positive impact on the social skills that facilitate interpersonal relationships (12, 13).

Therefore, the alternative of integration in regular employment is the only one that can fulfill the criteria of normalization and integration. Still, this social group presents very low labor integration rates despite their desire to work due to lack of opportunities emanating from public policies deficiencies and unavailability of clear variables influencing job access and retention by people with SMDs which complicates the development of final conclusions (14).

The Individual Placement and Support (IPS) model is a solid approach based on scientific evidence that helps people with SMDs quickly find employment, meeting each individual’s occupational needs and preferences, while providing flexible and ongoing support during and after attaining employment, in cooperation with the public health care system. Previous research supports IPS as a highly effective model to improve employment outcomes in individuals with SMDs, proving its wide applicability and positive impact on mental health recovery (15–19). IPS extension to populations outside the United States of America (USA) has been a relatively recent development. It has demonstrated its efficacy in high-income countries and is now expanding to new environments and countries with average incomes. (20), programs being carried out in up to 19 countries (Australia, Belgium, Canada, China, Czeck Republic, Denmark, France, Germany, Iceland, Ireland, Italy, Japan, New Zealand, the Netherlands, Norway, Spain, Sweden, Switzerland and the UK) (21).

Analysis of labor market in Spain reflects low participation of people with disabilities: 35.3% vs 78% of people without disabilities. Specific information on employment of people with SMDs is not included in national statistics, being subsumed into the general category of “mental disorders”. Thus, the lack of concrete data on employment of people with SMDs. People with “mental disorders” present an even lower participation in the labor market, 29.2%, compared with other disabilities (22). More specifically, in the Canary Islands, participation in the labor market of people with disabilities is even lower than other regions, with 24% vs 73.42% of people without disabilities. Moreover, the prevalence of disability due to “mental disorder” in this region is higher than that of the rest of the country with 34.4% vs 21.2% (23).

In this context, the Individualized Employment Support Team (Spanish acronym EAIE) of the Island Society for the Promotion of People with Disabilities (Spanish acronym SINPROMI) has been working on the island of Tenerife since 2004 toward job integration of people with SMDs following scientific evidence using IPS model and providing individualized and specialized care (24). Six employment specialists cover the whole territory in coordination with community multidisciplinary teams distributed across the territory, with whom they conduct regular follow-up meetings of users derived to employment specialists. These employment specialists are assigned a maximum of 25 people with SMDs (25). Fidelity to the IPS model is assessed using the fidelity scale for the IPS model of supported employment. This scale comprises 15 items, each with 5 response options, allowing for adequate differentiation between individualized employment support and other vocational interventions (26). An external professional conducted the IPS fidelity scale during the period immediately prior to the start of the study. The EAIE team scored 66 out of a possible 75 points, indicating that the program’s implementation is classified as “Good”.

This networking of EAIE, along with collaboration between different professionals, promotes vocational services and increases the effectiveness of the treatment and rehabilitation plan for various reasons. First, the addition of IPS to mental health treatment maximizes the chances that the client’s clinical treatment providers will support the client in pursuing his or her vocational goals. Second, issues pertinent to the clinical management of the client’s psychiatric disorder may become apparent to the IPS specialist in the process of working with the individual, and communicating these problems to the treatment team may lead to effective solutions that both address the clinical problems and increase the client’s ability to work. Third, clinicians involved in the treatment of the client’s psychiatric disorder may have valuable suggestions for job leads. Fourth, clinicians may help address problems that interfere with work, such as inadequate coping, relapses, substance abuse, and limited interpersonal skills by providing these treatments directly or referring clients to appropriate services (27).

Researchers agree on three main types of predictors: patients’ characteristics, environmental factors, and interventions (28). Adverse symptomatology and lack of social skills are hindering factors (29). Similarly, interpersonal difficulties constitute a primary factor in the termination of unsatisfactory jobs as they affect workplace behavior and confidence. The factors related to healthcare and treatment significantly impact the employment decisions (30). The following factors correlate positively with subsequent employment: job training and experience (31), desire to work (10), maintenance of cognitive functions (32), salary (33, 34) and the existence of natural supports (35).

One of the non-vocational variables linked to the employment outcomes of people with SMDs is level of satisfaction, understood as the positive or pleasurable emotional estate resulting from the person’s subjective perception of job experiences (36). The association between job match (the alignment between job preferences and the job obtained) and tenure in employment among individuals with Serious Mental Disorders has been studied. More specifically, high scores in interest and enjoyment show positive correlations with months worked, notably the item linked to boredom. Moreover, regarding perceived competence, pride in a job done well can be highlighted as an item associated with permanence in the job (37). These findings are consistent with studies highlighting significant correlation between job satisfaction and permanence in the job among people with SMDs (38).

Approaching this reality involves getting firsthand knowledge of the personal stories and how the subject himself, as their protagonist, interprets them. There is a reduced number of studies on work experiences by people with SMDs based on a qualitative approach from the methodological point of view (39–41). This study aimed at obtaining information on work experience and satisfaction in the workplace directly from the worker with SMDs.

## 2 Materials and methods

### 2.1 Sample

Group of workers with SMDs: The research team recruited a sample of 24 individuals diagnosed with SMDs, including mostly schizophrenia (10 subjects), psychosis of unknown origin (5 subjects), bipolar disorder (4 subjects), delusional disorder (3 subjects), and schizoaffective disorder (2 subjects). Community mental health teams on the island of Tenerife, including those from public mental health services and the Individualized Employment Support Team of the Island Society for the Promotion of the Disabled (SINPROMI), followed these individuals. This team specializes in integrating individuals with SMDs into the workforce using the IPS methodology.

We considered the following inclusion criteria: age between 18 and 65, having a diagnosis of serious mental disorders, being followed up by the mental health services, remaining stable at a psychopathological level, being willing and consenting to participate, and working at the time of the study. We excluded those who did not meet any inclusion criteria and those on sickness leave or temporarily unable to work.

Control group: We set up a control sample of 24 subjects. This control group consisted of matched pairs based on the same employer and job position. We considered the following inclusion criteria: working in the same company, performing the same work position, having held the position for the same time, having equal duration of the contract, having the same working hours, being of the same sex, age group, and level of studies, and declaring willingness to participate in the study and consenting to it. We excluded those presenting any kind of disability and those lacking willingness.

### 2.2 Type of study

We used a mixed qualitative-quantitative methodology. We employed thematic analysis of information as a qualitative strategy and the Indiana Job Satisfaction Scale (IJSS) as a quantitative tool to measure the level of job satisfaction, thus combining both methodologies.

### 2.3 Qualitative methodology

Semi-structured interview: We developed an interview protocol based on predictors of employment for people with SMDs included in scientific literature (42, 43). Thus, we created a 40-question semi-structured interview (**Supplementary Material**).

Procedure: We performed purposive sampling based on the available voluntary cases. Sampling was sequential, meaning saturation guided the creation of the sample, and we made decisions as concepts emerged.

We carried out the interview individually with workers with SMDs, and we electronically recorded the audio and registered it with prior informed consent. We then reviewed the recordings and manually transcribed their literal content, reflecting the declarations as accurately as possible. We later analyzed the information obtained using NVIVO text analysis software by importing transcriptions, and categorizing and codifying the information, thus facilitating pattern analysis and exploration within the data. We considered the frequency of word emergence when analyzing the content of the interviews, assuming the importance of their recording increases with their frequency of emergence.

A specialist in the study theme simultaneously conducted a general revision of data and coding, allowing cross-analysis and enriching the initial thematic analysis to compensate the potential bias of the said analysis performed from a unique perspective and as an internal validity criterion.

### 2.4 Quantitative methodology

The Indiana Job Satisfaction Scale: We used the IJSS as a measuring tool to assess the level of job satisfaction of the subjects at the time of the individual interview. It is a self-report scale with 32 items divided into a total of 6 subscales (38). Data on the scale’s reliability show an internal consistency, Cronbach’s alpha, of.90 for the total scale, and internal consistency coefficients ranging from a maximum of.83 (Work colleagues) to a minimum of.41 (Promotion and Safety) for the subscales, as confirmed by the Chinese version of the scale (44), which has acceptable psychometric properties.

Procedure: We applied the IJSS to the group of workers with SMD. As an SMD worker joined the study, we contacted their counterpart worker without disability and invited them to join the study. Subsequently, we applied the IJSS to the counterpart, thus creating the control group.

Despite the limitations inherent in performing inferential analyses with small sample sizes, we conducted a thorough analysis for each demographic variable considered. We compared individuals with SMDs to the control group for each demographic variable using the chi-square test (*X* ^2^).

Furthermore, we executed a matched case-control analysis for the variables of sex, age, work hours, job position, and salary. For numerical variables, we applied the Student’s t-test for paired samples to identify any significant differences between the two groups. For nominal variables, we evaluated both raw concordance and chance-adjusted concordance using Cohen’s Kappa statistic to verify the matching process.

We assessed the normality of the variables using the Kolmogorov-Smirnov test prior to analysis. Additionally, with respect to the satisfaction scores across the six IJSS subscales, we performed a matched case-control analysis using the Student’s t-test for paired samples and examined each item with the Wilcoxon signed-rank test.

## 3 Results

**Table 1** shows the characteristics of the samples, highlighting the similarities between both samples of participants, as well as some actual differences due to participants’ availability. Additionally, we observe the scores obtained from the chi-square tests (*X* ^2^) in relation to the differences in the variables.

**Table 1:** Characteristics of the sample.

		SMD Group	Control Group	P (<0.05)
**Sex**	Man	18	16	0.751
	Woman	6	8	
**Age**	20-49 years	16	18	0.751
	50-65 years	8	6	
**Educational Level**	Primary Education	13	9	0.152
	Compulsory Secondary Education	2	2	
	Vocational Training	5	7	
	High School Studies	1	5	
	Higher Education	3	0	
**Work Positions**	Assistant waiter	6	6	1.000
	Room attendant	3	3	
	Cleaning staff	3	3	
	Factory worker	2	2	
	Construction laborer	2	2	
	Administrative assistant	1	1	
	Entry-level technician	1	1	
	Garden assistant	1	1	
	Storekeeper	1	1	
	General helper	1	1	
	Porter	1	1	
**Working hours**	Full time	17	18	1.000
	Part time	7	6	
**Contract duration**	Temporary	13	4	0.016
	Indefinite	11	20	
**Time in the position**	Less than a year	15	9	0.149
	More than a year	9	15	

Regarding the economic benefits, 11 subjects were not receiving any economic benefits, while 13 received some kind of benefit.

### 3.1 Analysis of qualitative content: interviews

We obtained results through the analysis of qualitative data collected from 24 interviews with SMD workers, each lasting an average of 13 minutes.

We defined the codes obtained from the interview information as closely as possible to the statements made. We identified four main types of information covering the remaining codes. The hierarchical map of coding shows increased coding in the Individual Predictor category, followed by the categories of Environmental Predictors, Social Predictors, and External Factors. This indicates that the narratives focused on characteristics directly affecting the worker and influencing the performance of the tasks assigned to the position.

In the Individual Predictors category, the coding highlights the psychological function of employment, as well as self-esteem and self-confidence. Regarding the psychological function of employment, testimonies emphasize motivation, understood as the reasons why the person wants to have a job. Participants prioritize economic motivation, interpersonal relationships, and the opportunity to build a life project. Regarding information on self-esteem and self-confidence, the narratives focus on how participants value their own capacities and self-esteem. This coding materializes in self-fulfillment codes (achievement of aspirations or life goals) and satisfaction with good performance (perception of satisfaction with the work performed).

Individual predictors//psychological function//economic motivation:

- *It’s the main thing for me! Whoever says he enjoys working for the fun of it is lying! People work for money, that is that. You cannot live without money.*

- *Mainly economic, to be, to help at home with the bills, to help my mother.*

Individual predictors//psychological function//interpersonal relationships:

- *I like it because it gives me a challenge and an environment where I deal with people all day and … and you learn to deal with people and in your department, as I was saying, you get to see a lot of people, you share things and that is fine.*

Individual predictors//psychological function//life project:

- *I can easily take, if I get pregnant in the future, I can easily take maternity leave … I can take leave and rest assured.*

- *Also, the issue of unemployment contribution … that is, for … for the benefits, to get benefits in the future, mainly that.*

Participants identified satisfaction with good performance and the feeling of self-fulfillment through the achievement of aspirations or life goals as the main factors that reinforce self-esteem and self-confidence.

Individual predictors//self-esteem/self-confidence//satisfaction with good performance:

- *Level is quite good, you see, when you finish your day’s work, it’s rewarding, you see, I’ve done it … everything work related is well done, finished, that’s something good.*

Regarding Environmental Predictors, people with SMDs expressed that they find their work physically hard. However, on a psychic level, they generally consider employment to be beneficial, despite perceiving certain negative impacts due to stress caused by workload, working pace, and dealing with customers. They declared satisfaction with their working hours, mostly conducting varied tasks in teams, and generally felt well paid for their work. Few promotion opportunities or related prospects for improvement are perceived. Continuity and permanence in the job are highly appreciated, with special value attached to indefinite contracts, and there is concern about the approaching end of contracts.

Environmental predictors//characteristics of the position//physically hard:

- *Well, it makes me feel … well, a bit tired, but man! comes Thursday and I already feel a bit more tired, you know? a bit lower as the week passes.*

- *Environmental predictors//characteristics of the position//speed is required:*

- *It depends on the day, some days they rush you.*

- *To be quick a little, to rush a little, to hurry. To hurry up a little, you sweat a little and so…*

Environmental predictors//characteristics of the position//it affects on a psychic level:

- *It’s also hard psychically. Because, I don’t know,… tension accumulates.*

- *I try not to overheat because I overheated at the beginning. Now I try not to get angry or overheat or …*

- *Sometimes, yes, sometimes, for example, before starting to work, I, personally, get a bit anxious. “How is it going to go? Oh my God! I have to be there for four hours!” … so, tense. But as I start to work it passes, it gets better.*

- *The stress of work. The moment I leave work and get home I look for activities to lose my inhibitions or take away the stress, you see?*

- *On a psychic level, it is … it stresses you out a little at the beginning because you must be … dealing with clients was not my thing, but you get … you keep seeing people and you get to almost anticipate the difficulties they might cause.*

Environmental predictors//characteristics of the position//on a psychic level//brings benefit:

- *I feel this has a positive impact on us, let’s say, making our brains work a little harder, exercising our memory.*

- *It’s going to make you pull your socks up, because on a psychological level, memory … What does your brain do? Well, it has to work automatically, and this is exercising the brain, it is then psychologically positive, very, very positive.*

- *Good! (.) I think it’s good. I … I think it keeps you well (.) keeping yourself busy and active is something good. Whenever you go from doing something to not doing anything or from not doing anything to doing something, these are changes that. well … shake any psychic structure.*

Environmental predictors//characteristics of the position//satisfaction with salary:

- *Well, my salary is quite ok for my professional category compared to other places. To tell the truth, salary is quite good.*

- *Salary is obviously good. The job is, let me tell you, it is challenging. It is hard at times, but the salary makes up for that. You could say it compensates my labor.*

Environmental predictors//characteristics of the position//romotion:

- *No, not here. Here, Mr X already told me one day that here … it is going to be always the same. This is outrageous, it discourages people. Not letting people better themselves, but instead saying … it’s like a life sentence. You aren’t going to redeem yourself; you remain the same.*

- *I have given it a thought sometimes, but I don’t see it happening. I am the only disabled worker in the company, so I’m not going to be the one promoted, it’s not going to be me.*

Among Social Predictors, relations with work colleagues and supervisors play a key role. Information reflects that relationships with work colleagues are good and focused mainly on the workplace. Relationships with supervisors are also considered good in most cases and occur both in and outside of work, with shared moments and situations outside working hours. Interaction with the person directly responsible for the position is also considered positive, though this relationship is limited to the workplace. The treatment received from the company is considered good, and its feedback is positive, providing information on performance. Regarding prejudice and discrimination detected by SMD workers, they distinguish between disabilities due to mental health disorders and other types of disabilities. Workers with mental health disorders detect increased employment difficulties owing to the type and characteristics of their disability. Specifically, the fact that psychic disabilities are less visible than physical ones makes some work colleagues, who know about the disability but not the exact diagnosis, feel curious and suspicious, causing SMD workers to perceive increased discrimination.

Social predictors//prejudice-discrimination//mental health vs. other disabilities:

- *This has to do with the stigmatization of our illness. Mainly, we have to … let’s make a … a … a separate chapter for the psychically disabled.*

- *The disadvantage lies in the fact that people who see me do not understand exactly which is my disability, and why I’m here, and why … and he asks, sometimes they ask and don’t discriminate me. On certain matters … some people don’t discriminate me, others do.*

- *The psychically and intellectually disabled are hit much harder by this kind of things than … a person like … who has … with a missing finger, or hand … I don’t know. His disabilities are built-in [he/she laughs] you can see them. In our case, however, our … psychic disability, we can seem normal people the way … psychiatry considers us now. We can seem normal people, if you are listening to me, if you are listening to me right now you may think that I am thinking, that I am elaborating thoughts, that, maybe, I don’t know … (.) it would be crazy to think I could, I could do it. Then, my work colleagues, of course I understand them “What is his/her disability? [he/she laughs] I cannot see any disability, why is he/she here?”. It is then normal to a certain point, it’s understandable them saying so. It’s understandable that our work colleagues don’t feel ok, no, no … I mean no…*

Social predictors//good relationship with work colleagues//outside workplace:

- *Yes, yes, with “A”. Sometimes, not very often, but from time to time we go for a beer after work to a bar near the company, and then we go home.*

Social predictors//relationship with superior//positive feedback:

- *He told me to … to go on like this and … not to … and to carry on in the same line and to … keep working like this, you know? Not to rest on my laurels or so.*

- *Yes, yes, yes, yes. He/She congratulates me a lot and very often. He/she comes and tells me … pats me on the back: Hey, you did well today! You did really well today. Yes, always, always.*

The external factors identified in the participants’ experiences refer to agents who may cause a positive or negative impact on the working experience and over whom the organization has no control. Participants identified clients and considered interactions with them to be positive. They also identified families, most of whom value positively that their family member is working without their mental health condition being considered a limitation and appreciate the benefits employment brings. Among the negative opinions, family members often considered their relatives to be overqualified for the job they performed.

External factors//clients:

- *They are really happy with me, and the boss even writes e-mails congratulating me because clients are grateful about how I treat them and the information I give them and how approachable and respectful I am, and everything I provide them, everyone loves.*

External factors//family//positive opinion:

- *They were happy that I had started working and encouraged me even more. “Yes, that’s good … well done, go on. You need to work, go out a bit … get your mind off things. Because it helps (with your recovery).” Yes, yes. I get a lot of advice from them.*

External factors//family//negative opinion:

- *They tell me ‘‘well, how can you be working as a cleaner with your formation? and so…’’*

### 3.2 Analysis of quantitative content: IJSS

The matched case-control analysis of the demographic variables in the sample yielded the following results:

For the variable *sex*, Cohen’s Kappa test revealed a raw concordance of 92% and a chance-adjusted concordance of 80%, with the latter being statistically significant (p < 0.001).

The analysis of *age* using the Student’s t-test for paired samples showed no significant difference between the mean age of individuals with SMDs (M=45.08; SD=11.10) and the mean age of the control group (M=42.04; SD=10.82; t_(23)=_0.96, p=0.34).

Regarding *work hours*, Cohen’s Kappa test demonstrated a high level of similarity between the two samples. The results showed a raw concordance of 88% and a net concordance of 68%, which was statistically significant (p< 0.001).

For the *job position*, the raw concordance between the samples was 100%, and the net concordance was 95%, both statistically significant (p < 0.001). This indicates that individuals with SMDs held the same job positions as those in the control group.

In terms of *salary*, categorized into those earning more than 1000 euros and those earning less than 1000 euros, Cohen’s Kappa test reflected a raw concordance of 88% and a net concordance of 73%, which was statistically significant (p < 0.001).

The data on *contract type* revealed a higher percentage of temporary contracts (54%) versus permanent contracts (37%) in the SMDs group. In contrast, the control group had 71% permanent contracts and 17% temporary contracts. The raw concordance obtained using Cohen’s Kappa was 50% and the net concordance was 21%, which was not statistically significant (p > 0.001). This result indicates a notable discrepancy in hiring patterns between individuals with SMDs and the control group. The McNemar-Bowker test confirmed this with a statistically significant difference (p < 0.05) in contract type (temporary vs. permanent) between the SMDs group and the control group.

For the variable *time in position*, categorized as >1 year or <1 year, Cohen’s Kappa test yielded a raw concordance of 54% and a net concordance of 12%, which was not statistically significant (p > 0.001). This suggests that individuals with SMDs stay in their positions for a shorter duration compared to the control group.

Regarding the *overall average score* on the IJSS scale, the results indicate similar levels of satisfaction between the two samples. The Student’s t-test for paired samples determined no significant differences in the overall average satisfaction score for the SMDs group (M=3.21; SD=0.32) compared to the control group (M=3.16; SD=0.41; t_(23)=_0.45, p=0.66).

As shown in **Table 2**, the analysis did not reveal statistically significant differences across any of the six IJSS dimensions between the SMDs group and the control group.

**Table 2:** Measurements, means and difference test for the different IJSS subscales.

SUBSCALE	GROUP	MEANS	SD	t (23)	p
General satisfaction	SMD	3.46	.47	.44	0.66
	Control	3.40	.52		
Salary	SMD	1.67	.48	.60	0.58
	Control	1.63	.49		
Promotion and stability	SMD	2.99	.52	.08	0.94
	Control	3.00	.60		
Supervision	SMD	3.23	.48	.09	0.93
	Control	3.22	.62		
Work colleagues	SMD	3.13	.52	1.19	0.25
	Control	3.32	.51		
How do I feel at work?	SMD	3.11	.43	1.31	0.20
	Control	2.90	.52		

The analysis of the IJSS items using the Wilcoxon signed-rank test revealed a statistically significant difference (p < 0.05) between individuals with SMDs and the control group for the item *“I need more money than I am paid for this job”* (Z = -1.97, p = 0.04). This result suggests that individuals with SMDs report a higher need for additional income compared to those in the control group.

Additionally, we observed a statistically significant difference (p < 0.05) in the perception of *“having a coworker whom I consider a friend”* between the SMDs group and the control group (Z = -2.66, p = 0.008). This finding indicates that individuals with SMDs report having fewer coworkers they consider friends compared to the control group. No statistically significant differences were found for the other items analyzed.

## 4 Discussion

For most people sharing their recovery experiences, employment plays a key role in the process (45). Thus, it is important to understand the meaning and satisfaction workers with SMDs perceive during their experiences in regular jobs.

We conducted this study on a sample of 24 workers with SMDs who found jobs through the Individualized Employment Support Team, which has been promoting labor integration of people with SMDs since 2004 using the IPS model on the island of Tenerife (Spain), the largest and most populous of the Canary Islands with over 900,000 inhabitants.

The results of this study show the importance of understanding subjective well-being through personal descriptions of each case, to grasp the complexity and uniqueness of individual recovery processes of control and sense of personal desires by people with SMDs.

From both qualitative and quantitative perspectives, we found a high degree of job satisfaction and a similarly positive job evaluation by SMD workers and other workers. Analysis of experiences in regular employment of each participant confirms the results obtained in previous similar research, identifying both facilitators and barriers in the shared realities (6, 46, 47).

We performed the analysis of qualitative information using Nvivo software, assuming that the significance of a registration unit grows with its frequency of occurrence. We compared data with data and, subsequently, with codes using the constant comparative method (48), refining and deepening the analysis, which led to the emergence of new codes, some of which reached the category level.

Participants identified key facilitators within environmental predictors, particularly their assessment of the psychic characteristics of the job position (understood in coding as the effects of the psychological demands of the job). They perceived that the job did not interfere in this domain and sometimes even brought benefits, such as cognitive function improvement and distraction by keeping the mind busy. Regarding stress, people with SMDs perceived an increase in stress levels within the work environment. However, testimonies indicated that this stress was primarily associated with specific situations, such as starting a new job or facing new tasks. This stress manifested exclusively within the work context and was managed without difficulty, not extending to other areas of life outside of work. This finding contradicts the current controversy in the literature regarding the connection between stress and the onset and progression of schizophrenia within the framework of the stress vulnerability model (49). In fact, none of the participants required hospitalization during their contract, using this indicator to identify the most acute stress.

Moreover, within the psychological function of employment, participants’ narratives highlighted improvements in mental health due to the self-perception of a better mood and the opportunity employment brings to keep the mind busy and entertained.

However, participants perceived a barrier regarding the physical characteristics of the position, noting that the tasks performed required great physical effort. Literature shows that people with SMDs typically obtain low-skilled positions (50).

When accessing employment through the EAIE, the employment specialist only informed the employer of the disability, not disclosing the mental health diagnosis, as it is a personal decision for the worker with SMDs. None of the sample workers with SMDs chose to share such information, so the IPS specialist worked “behind the scenes” to help the worker reach their goals, such as negotiating reasonable adaptations with the employer. Participants expressed increased difficulties in getting a job depending on their type of disability and mentioned workplace situations conditioned by prejudice or discrimination, differentiating between mental health-related disabilities and other types of disabilities. The invisibility of psychic disabilities compared to physical disabilities led some work colleagues, who knew about the disability but not the specific diagnosis, to be curious and cautious, causing SMD workers to perceive increased discrimination. These findings align with other research highlighting the fear of discrimination experienced by people with SMDs when revealing information about their disorder in job interviews, thinking it might hinder their employment opportunities (51). This is relevant to the employment strategy used, in this case, IPS, which is based on integration into regular employment and standardized working environments.

In agreement with literature (33), participants emphasized the importance of salary and their satisfaction with it, considering it a motivating factor. The significance of the economic factor is also reflected in the IJSS scores, which reveal a significant difference for the item *“I need more money than I am paid for this job”.*Participants also mentioned interpersonal relationships in the workplace, both with work colleagues and supervisors, as a factor promoting job satisfaction. They referred to positive experiences that extended beyond the workplace in the case of work colleagues, which helped widen their social network. Previous research has shown this positive impact on social functioning (52).

Participants considered making a career in the companies through promotion unlikely, reflecting low expectations of improvement. Given the opportunity, they were reluctant to acquire further responsibilities in the position. Previous research shows that the employment preferences of people with SMDs are realistic and stable over time (34). Participants voiced concerns about the temporality and termination of employment contracts, noting that these factors can disrupt their personal future planning. The demographic analysis revealed that individuals with SMDs are more likely to have temporary contracts compared to the control group, suggesting a potential discrimination or inequality in access to more stable employment opportunities for those with SMDs. Although there was observed concordance, the lack of statistical significance implies that these results may be subject to variability, highlighting the need for further research to confirm these findings and better understand the underlying causes of these discrepancies in contract types. Consistent with this, the variable “time in position” also indicates shorter job tenure among workers with SMDs. The shorter job tenure and the temporary nature of the contracts, in this context, refer to employment contracts that end with one company but may be followed immediately by new employment with another company. This result in a series of contracts that allow workers with SMDs to remain actively employed. Membership in the IPS team facilitates the ability of individuals with SMDs to find new employment opportunities. Alternatively, this phenomenon may be related to labor legislation, employment policies in the service sector, and the preferences of individuals with SMDs. Previous studies show brief job tenure among people with SMDs; however, job tenure significantly increases among employed individuals with SMDs when high-fidelity IPS is implemented (53). This improvement reflects a strong commitment to the IPS principle of achieving competitive employment.

Accordingly, IJSS results indicate a significant relationship between employment tenure and how the subject feels in the job, with satisfaction being higher among workers with SMDs who had kept the job for more than a year when asked ‘how do I feel at work?’

Workers generally reported that their integration into society through obtaining regular employment is well valued by their families, who provide support for people with SMDs. Previous studies have highlighted this support from key people as a driving force that gives meaning to their role as workers (54).

However, among the negative opinions of family or friends is the perception that the worker performs tasks below their capacities. As shown in **Table 1**, the study participants occupy low-skilled jobs. Most participants have primary education, and their work profiles match the positions they hold. This corresponds with national statistical data, which show increased recruitment in low-skilled positions and lower educational levels among working people with disabilities (22). This suggests that families’ expectations may be unrealistic.

In this case, combining both methodological strategies clarified the meanings and verified the consistency of the obtained information. However, it is important to note that the generalizability of these findings may be limited due to the highly selective sample. The researcher who conducted the interviews was an employment specialist and a member of the EAIE team. This could introduce a potential bias in the qualitative aspect of the study, for which a parallel review by an expert was implemented as a control measure. However, recent studies on qualitative research highlight the researcher as the primary data collection instrument and argue that the strengths of qualitative data, such as their richness, can offset the subjectivity and limitations in generalizing results (55).

Additionally, differences in some variables and characteristics of the subjects from both groups due to participants’ availability may exist despite considering inclusion criteria. The lack of a second measure of evaluation with IJSS in the sample of workers with SMDs implies a limitation in obtaining information on the evolution of satisfaction levels over time.

In conclusion, job satisfaction among people with SMDs is similar to that of other workers and stands out as a key factor in retaining a job and promoting individual recovery processes.

## Data Availability

The original contributions presented in the study are included in the article/**Supplementary Material**. Further inquiries can be directed to the corresponding author.

## References

[1] Goldman HH Gattozzi AA Taube CC. Defining and counting the chronically mentally ill. Hosp Community Psychiatry. (1981) 32:21–7. doi: 10.1176/ps.32.1.21 7461614

[2] National Institute of Mental Health. Towards a model for a comprehensive community based mental health system. Washington DC: NIMH (1987).

[3] Valiente Ots C Espinosa López R. What is severe mental disorder? EduPsykhé: J Psychol Psychoped. (2017). 16(1):4–14.

[4] Drake RE Wallach MA. Employment is a critical mental health intervention. Epidemiol Psychiatr Sci. (2020) 29:e178. doi: 10.1017/S2045796020000906 33148366 PMC7681163

[5] Rüsch N Nordt C Kawohl W Brantschen E Bärtsch B Müller M. Work-related discrimination and change in self-stigma among people with mental illness during supported employment. Psychiatr Serv. (2014) 65:1496–8. doi: 10.1176/appi.ps.201400073 25270160

[6] Dunn EC Wewiorski NJ Rogers ES. A qualitative investigation of individual and contextual factors associated with vocational recovery among people with serious mental illness. Am J Orthopsychiatry. (2010) 80:185–94. doi: 10.1111/j.1939-0025.2010.01022.x 20553512

[7] Rodríguez Pulido F Rodríguez Díaz MN. The importance of job support in the recovery of individuals with severe mental disorders. Ment Health. (2013) 36:37–42. doi: 10.17711/SM.0185-3325.2013.005

[8] Millner UC Rogers ES Bloch P Costa W Pritchett S Woods T. Analyzing the meaning of work for individuals living with serious mental illnesses. J Prof Dev. (2022) 49:393–410. doi: 10.1177/0894845320941256

[9] Agencia Estatal Boletín Oficial del Estado. Supported employment program as a measure to promote employment for people with disabilities in the mainstream labor market (2007). Available online at: https://www.boe.es/eli/es/rd/2007/07/02/870.

[10] Gühne U Pabst A Löbner M Breilmann J Hasan A Falkai P. Employment status and desire for work in severe mental illness: results from an observational, cross-sectional study. Soc Psychiatry Psychiatr Epidemiol. (2021) 56:1657–67. doi: 10.1007/s00127-021-02088-8 PMC8429146 33860804

[11] Adamus C Richter D Sutor K Zürcher SJ Mötteli S. Preference for competitive employment in people with mental disorders: A systematic review and meta-analysis of proportions. J Occup Rehabil. (2024) 34:1–20. doi: 10.1007/s10926-024-10192-0 PMC12089194 38662329

[12] McDonald-Wilson KL Rogers S. An investigation of reasonable workplace accommodations for people with psychiatric disabilities: quantitative findings from a multi-site study. Community Ment Health J. (2002) 38:35–50. doi: 10.1023/A:1013955830779 11892855

[13] Gowdy EL Carlson LS y Rapp CA. Practices differentiating high-performing from low-performing supported employment programs. Psychiatr Rehabil J. (2003) 26:232–9. doi: 10.2975/26.2003.232.239 12653445

[14] López Menéndez LA. Predictors of work performance in individuals with severe mental disorders. Rev Stud evidence analysis Psychosocial Intervention. (2008) 17:245–68. doi: 10.5093/in2008v17n3a2

[15] Bond GR Drake RE. Making the case for IPS supported employment. Administration Policy Ment Health Ment Health Serv Res. (2014) 41:69–73. doi: 10.1007/s10488-012-0444-6 23161326

[16] Reme SE Monstad K Fyhn T Sveinsdottir V Løvvik C Lie SA. A randomized controlled multicenter trial of individual placement and support for patients with moderate-to-severe mental illness. Scand J Work Environ Health. (2019) 45:33–41. doi: 10.5271/sjweh.3753 30074050

[17] de Winter L Couwenbergh C van Weeghel J Sanches S Michon H Bond GR. Who benefits from individual placement and support? A meta-analysis. Epidemiol Psychiatr Sci. (2022) 31:1–24. doi: 10.1017/S2045796022000300 PMC9281491 35815640

[18] Christensen TN Wallstrøm IG Stenager E Hellström L Bojesen AB Nordentoft M. 30-month follow-up of individual placement and support (IPS) and cognitive remediation for people with severe mental illness: Results from a randomized clinical trial. Psychiatry J. (2023) 1–10. doi: 10.1155/2023/2789891 PMC10162865 37151719

[19] Bond GR Al-Abdulmunem M Marbacher J Christensen TN Sveinsdottir V Drake RE. A systematic review and meta-analysis of IPS supported employment for young adults with mental health conditions. Adm Policy Ment Health. (2023) 50:160–72. doi: 10.1007/s10488-022-01228-9 PMC10221953 36219318

[20] Drake RE Bond GR. Individual placement and support: history, current status, and future directions. PCN Rep. (2023) 2:1–8. doi: 10.1002/pcn5.122 PMC11114326 38867819

[21] Drake RE. Special issue: implementation of individual placement and support. Psychiatr Rehabil J. (2020) 43:1–82. doi: 10.1037/prj0000401 32068428

[22] National Institute of Statistics. Employment of people with disabilities (2023). Available online at: https://www.ine.es/prensa/epd_2022.pdf.

[23] Observatorio Canario de Empleo. Report on the Labor Market for People with Disabilities (2022). Available online at: https://drive.google.com/file/d/1a6JQd7i7m8T9nWUNqf9DjISNWwcTwJUg/view?pli=1.

[24] Rodríguez Pulido F Caballero Estebaranz N Aldana E Abad M Álvarez-Sotomayor M Reig S. Effectiveness of an individualized employment support strategy for people with severe mental disorders. Gaceta Sanitaria. (2018) 32:513–8. doi: 10.1016/j.gaceta.2017.05.007 28712681

[25] Rodríguez Pulido F Caballero Estebaranz N Tallo Aldana E Hernández Álvarez de Sotomayor MC Vílchez de León PI López Reig S. The development in Tenerife of the Individualized Employment Support Teams (EAIE). Personal autonomy in regular employment for individuals with severe mental disorders. Sinpromi. (2011) 4):75–84. Available at: https://sinpromi.es/wpcontent/uploads/2021/01/librolaautonomiapersonalempleoordinario.pdf.

[26] Bond GR Becker DR Drake RE Vogler KM. A fidelity scale for the Individual Placement and Support model of supported employment. Rehabil Couns Bull. (1997) 40:265–84.

[27] Bond GR Mueser KT. Supported employment. In: Sowers WE McQuistion HL Ranz JM Feldman JM Runnels PS, editors. Handbook of Community Psychiatry, 2nd ed. Springer, Cham, Switzerland (2022). p. 513–24. doi: 10.1007/978-3-031-10239-4_37

[28] Bond GR Drake RE. Predictors of competitive employment among patients with schizophrenia. Curr Opin Psychiatry. (2008) 21:362–9. doi: 10.1097/YCO.0b013e328300eb0e 18520741

[29] Rodríguez Pulido F Rodríguez Díaz MN. Work performance and psychotic symptoms in individuals with severe mental disorders. Span Psychiatry Proc. (2015) 43:192–9. doi: 10.4321/S1139-92872015000500002

[30] Ramsay CE Stewart T Compton MT. Unemployment among patients with newly diagnosed first-episode psychosis: prevalence and clinical correlates in a US sample. Soc Psychiatry Psychiatr Epidemiol. (2012) 47:797–803. doi: 10.1007/s00127-011-0386-4 21541697

[31] Koletsi M Niersman A van Busschbach JT Catty J Becker T Burns T. Working with mental health problems: clients’ experiences of IPS, vocational rehabilitation and employment. Soc Psychiatry Psychiatr Epidemiol. (2009) 44:961–70. doi: 10.1007/s00127-009-0017-5 19280083

[32] Rodríguez Pulido F Caballero Estebaranz N González- Dávila E Melián Cartaya MJ. Cognitive remediation to improve the vocational outcomes of people with severe mental illness. Neuropsychol Rehab. (2019) 31:1–23. doi: 10.1080/09602011.2019.1692671 31752647

[33] Bryson G Lysaker P Bell M. Quality of life benefits of paid work activity in schizophrenia. Schizophr Bull. (2002) 28:249–57. doi: 10.1093/oxfordjournals.schbul.a006935 12693431

[34] Mueser KT Clark RE Haines M Drake RE McHugo GJ Bond GR. The Hartford study of supported employment for severe mental illness. J Consulting Clin Psychol. (2004) 72:479–90. doi: 10.1037/0022-006X.72.3.479 15279531

[35] Williams AE Fossey E Corbière M Paluch T Harvey C. Work participation for people with severe mental illnesses: An integrative review of factors impacting job tenure. Aust Occup Ther J. (2016) 63:65–85. doi: 10.1111/1440-1630.12237 26992084

[36] Locke E. The nature and causes of job satisfaction. In: Dunnette M, editor. Handbook of industrial and organizational psychology. Rand Mc Nally, Chicago (2009). p. 1297–349.

[37] Kukla M Bond G. Job match and job tenure in persons with severe mental illness. J Rehabil. (2012) 78:11–5.

[38] Resnick SG Bond G. The Indiana Job Satisfaction Scale: Job satisfaction in vocational rehabilitation for people with severe mental illness. Psychiatr Rehabil J. (2001) 25:12–9. doi: 10.1037/h0095055 11529447

[39] Moen EA Walseth T Larsen B. Experiences of participating in individual placement and support: A meta-ethnographic review and synthesis of qualitative studies. (2021) 35:736–50. doi: 10.1111/scs.12848 32271470

[40] Rodríguez Pulido F Melián Cartaya MJ Caballero Estebaranz N. Considerations on job satisfaction as a predictor of employment success for people with severe mental illness. J Schizophr Res. (2021) 7:1039. 10.1080/09602011.2019.1692671 31752647

[41] Lettieri A Díez E Soto-Pérez F Bernate-Navarro M. Employment-related barriers and facilitators for people with psychiatric disabilities in Spain. Work. (20222022) 71:901–15. doi: 10.3233/WOR-213642 35253720

[42] Lettieri A Díez E. A systematization of the international evidence related to labor inclusion barriers and facilitators for people with mental illness. A review of reviews. Sociologica Ital J sociology line. (2017) 3:5–5. doi: 10.2383/89515

[43] Finol O Perdigón A López V Carralón S Fraguas D. Relationship between employment and emotional state in people with severe mental disorder. J Spanish Assoc Neuropsychiatry. (2021) 41:25–32. doi: 10.4321/s0211-57352021000200006

[44] Tsang HW Wong A. Development and validation of the Chinese version of the Indiana job satisfaction scale (CV-IJSS) for people with mental illness. Int J Soc Psychiatry. (2005) 51:177–91. doi: 10.1177/0020764005056766 16048246

[45] Khalaf Beigi M Mohammadi Shahbolaghi F Rassafiani M Haghgoo HA Taherkhani H. The meaning of work in people with severe mental illness (SMI) in Iran. Med J Islamic Republic Iran. (2015) 29:179. PMC4431441 26034732

[46] Campbell K Bond GR Drake RE McHugo GJ Xie H. Client predictors of employment outcomes in high-fidelity supported employment: A regression analysis. J Nervous Ment Dis. (2010) 198:556–63. doi: 10.1097/NMD.0b013e3181ea1e53 20699720

[47] Viering S Jäger M Kawohl W. What factors influence the success of supported employment? Psychiatrische Praxis. (2015) 42:299–308. doi: 10.1055/s-0034-1387695 26308455

[48] Contreras Cuentas M Paramo-Morales D Rojano Alvarado Y. The grounded theory as a theoretical construction methodology. Rev Científica Pensamiento y Gestión. (2020) 47:283–306. doi: 10.14482/pege.47.9147

[49] Walker EF Trotman HD Pearce BD Addington J Cadenhead KS Cornblatt BA. Cortisol levels and risk for psychosis: Initial findings from the North American prodrome longitudinal study. Biol Psychiatry. (2013) 74:410–7. doi: 10.1016/j.biopsych.2013.02.016 PMC3707958 23562006

[50] Burns T Catty J Becker T Drake RE Fioritti A Knapp M. The effectiveness of supported employment for people with severe mental illness: a randomized controlled trial. Lancet. (2007) 370:1146–52. doi: 10.1016/S0140-6736(07)61516-5 17905167

[51] Cameron J Walker C Hart A Sadlo G Haslam I. Supporting workers with mental health problems to retain employment: Users’ experiences of a UK job retention project. Work. (2012) 42:461–71. doi: 10.3233/WOR-2012-1370 22523040

[52] Burns T Catty J White S Becker T Koletsi M Fioritti A. The impact of supported employment and working on clinical and social functioning: Results of an international study of individual placement and support. Schizophr Bull. (2009) 35:949–58. doi: 10.1093/schbul/sbn024 PMC2728809 18403375

[53] Bond GR Kukla M. Is job tenure brief in individual placement and support (IPS) employment programs? Psychiatr Serv. (2011) 62:950–3. doi: 10.1176/ps.62.8.pss6208_0950 21807836

[54] Kennedy-Jones M Joanne C Ellie F. Developing a worker role: stories of four people with mental illness. Aust Occup Ther J. (2005) 52:116–26. doi: 10.1111/j.1440-1630.2005.00475.x

[55] Borg C Muñoz Martín R. Naturalistic qualitative research (R. Muñoz, Trans.). In: Franco J Muñoz R, editors. Encyclopedia of Translation and Interpreting (ENTI). AIETI (2024), 315–24. Available at: https://www.um.edu.mt/library/oar/handle/123456789/123447.

