# Supplementary material for: Experiences of individuals with serious mental disorders in regular employment through the Individual Placement and Support model
Source: Front Psychiatry. 2024 Sep 10;15:1423742. doi: 10.3389/fpsyt.2024.1423742 (PMC11420022; doi:10.3389/fpsyt.2024.1423742)
Supplement: Supplementary file 1 [file DataSheet1.pdf]

frontiers

Supplementary Material

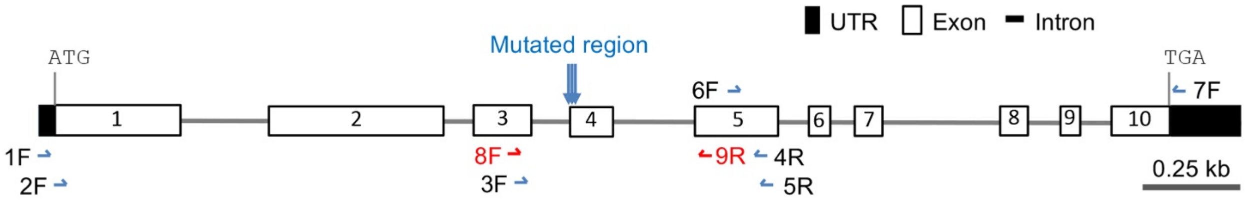

Supplementary Figure S1. Diagram showing the binding sites of *EGY1*-specific primers used to amplify *EGY1* cDNAs by RT-PCR. Relative positions of exons, introns, and the untranslated regions of the *EGY1* gene are represented. Primers 8F and 9R (marked in red) were used for next-generation sequencing analysis of splicing around the mutated region in *egy1-4* (marked in blue). For the information of primer sequences, refer to Supplementary Table S1.

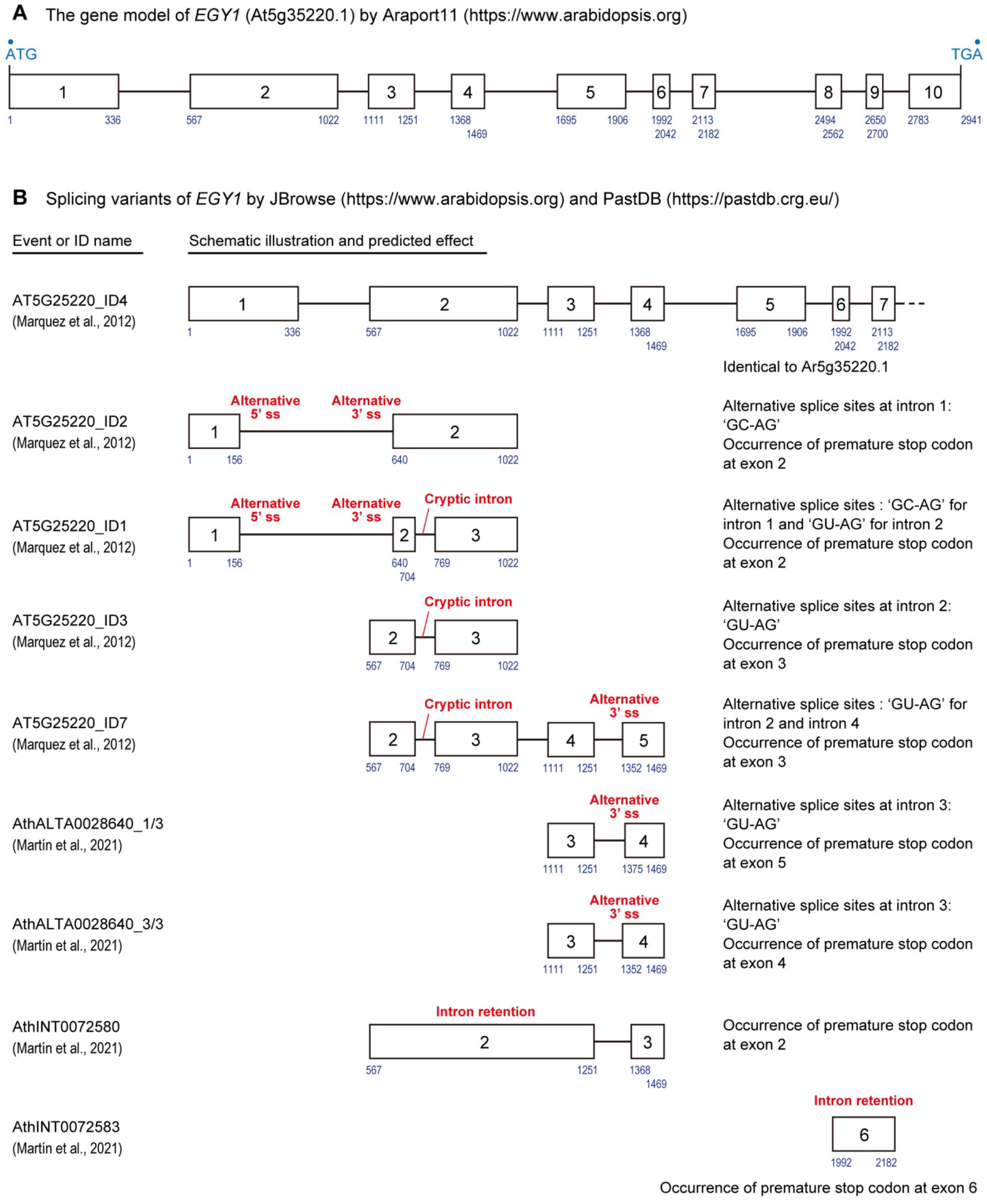

Supplementary Figure S2. Splicing variants of *EGY1* registered in public databases. (A) The gene model of *EGY1* in Araport11 (At5g35220.1; Chen et al., 2017). (B) Splicing variants of *EGY1* in TAIR (https://www.arabidopsis.org) and PastDB (https://pastdb.crg.eu/) web sites (accessed in Jul 2024). In (A) and (B), the number under boxes (exons) indicates the exon region at the genomic nucleotide level. The nucleotide 'A' in the start codon of *EGY1* was defined as 1. In (B), splicing variants determined by RNA-seq (Marquez et al., 2012; Martín et al., 2021) are shown and described.

```
Wild-type  MGTLTSVAFAAAVNIRFRSFHRENIKTTITTLPKWQKRLCFSSTEDSHRFRIAKCLGNDENSNRDDSIGENGETHKSSVVKTATFEEEDE  90
pattern 1  MGTLTSVAFAAAVNIRFRSFHRENIKTTITTLPKWQKRLCFSSTEDSHRFRIAKCLGNDENSNRDDSIGENGETHKSSVVKTATFEEEDE  90
pattern 2  MGTLTSVAFAAAVNIRFRSFHRENIKTTITTLPKWQKRLCFSSTEDSHRFRIAKCLGNDENSNRDDSIGENGETHKSSVVKTATFEEEDE  90
pattern 3  MGTLTSVAFAAAVNIRFRSFHRENIKTTITTLPKWQKRLCFSSTEDSHRFRIAKCLGNDENSNRDDSIGENGETHKSSVVKTATFEEEDE  90
pattern 4  MGTLTSVAFAAAVNIRFRSFHRENIKTTITTLPKWQKRLCFSSTEDSHRFRIAKCLGNDENSNRDDSIGENGETHKSSVVKTATFEEEDE  90
pattern 5  MGTLTSVAFAAAVNIRFRSFHRENIKTTITTLPKWQKRLCFSSTEDSHRFRIAKCLGNDENSNRDDSIGENGETHKSSVVKTATFEEEDE  90

                               intron 1                                              GNLR motif
Wild-type  ETSKSSSTTSSSNEFGSDKTSMPSTIDPTYSSFQIDSFKLMELLGPEKVDPADVKLIKDKLFGYSTFWVTKEEPFGDLGEGILFLGNLRG  180
pattern 1  ETSKSSSTTSSSNEFGSDKTSMPSTIDPTYSSFQIDSFKLMELLGPEKVDPADVKLIKDKLFGYSTFWVTKEEPFGDLGEGILFLGNLRG  180
pattern 2  ETSKSSSTTSSSNEFGSDKTSMPSTIDPTYSSFQIDSFKLMELLGPEKVDPADVKLIKDKLFGYSTFWVTKEEPFGDLGEGILFLGNLRG  180
pattern 3  ETSKSSSTTSSSNEFGSDKTSMPSTIDPTYSSFQIDSFKLMELLGPEKVDPADVKLIKDKLFGYSTFWVTKEEPFGDLGEGILFLGNLRG  180
pattern 4  ETSKSSSTTSSSNEFGSDKTSMPSTIDPTYSSFQIDSFKLMELLGPEKVDPADVKLIKDKLFGYSTFWVTKEEPFGDLGEGILFLGNLRG  180
pattern 5  ETSKSSSTTSSSNEFGSDKTSMPSTIDPTYSSFQIDSFKLMELLGPEKVDPADVKLIKDKLFGYSTFWVTKEEPFGDLGEGILFLGNLRG  180

                                                                                      intron 2
Wild-type  KKEDVFAKLQRKLVEVASDKYNLFMIEEPNSEGPDPRGGARVSFGLLRKEVSEPGPTTLWQYVIALILFLLTIGSSVELGIASQINRLPP  270
pattern 1  KKEDVFAKLQRKLVEVASDKYNLFMIEEPNSEGPDPRGGARVSFGLLRKEVSEPGPTTLWQYVIALILFLLTIGSSVELGIASQINRLPP  270
pattern 2  KKEDVFAKLQRKLVEVASDKYNLFMIEEPNSEGPDPRGGARVSFGLLRKEVSEPGPTTLWQYVIALILFLLTIGSSVELGIASQINRLPP  270
pattern 3  KKEDVFAKLQRKLVEVASDKYNLFMIEEPNSEGPDPRGGARVSFGLLRKEVSEPGPTTLWQYVIALILFLLTIGSSVELGIASQINRLPP  270
pattern 4  KKEDVFAKLQRKLVEVASDKYNLFMIEEPNSEGPDPRGGARVSFGLLRKEVSEPGPTTLWQYVIALILFLLTIGSSVELGIASQINRLPP  270
pattern 5  KKEDVFAKLQRKLVEVASDKYNLFMIEEPNSEGPDPRGGARVSFGLLRKEVSEPGPTTLWQYVIALILFLLTIGSSVELGIASQINRLPP  270
pattern 6  KKEDVFAKLQRKLVEVASDKYNLFMIEEPNSEGPDPRGGARVSFGLLRKEVSEPGPTTLWQYVIALILFLLTIGSSVELGIASQINRLPP  270
pattern 7  KKEDVFAKLQRKLVEVASDKYNLFMIEEPNSEGPDPRGGARVSFGLLRKEVSEPGPTTLWQYVIALILFLLTIGSSVELGIASQINRLPP  270
                                                                 TM helix 1
                                     intron 3  HEXXH motif                 intron 4
Wild-type  EVVKYFTDPNAVEPPDMELLYPFVDAALPLAYGVLGILLFHELGHFLAAVPKKVKLSIPYFIPNITLGSFGAITQFKSILPDRSTKVDIS  360
pattern 1  EVVKYFTDPNAVEPPDMELLYPFVDAALPLAYGVLGILLFHVSKNLCVSIVMLFFL----------------------------------  326
pattern 2  EVVKYFTDPNAVEPPDMELLYPFVDAALPLAYGVLGILLFHGNFLAAVPKKVKHSILHSKHYTRQLWGNHTV------------------  342
pattern 3  EVVKYFTDPNAVEPPDMELLYPFVDAALPLAYGVLGILLFHTCLFARKGELSCCSSKES-------------------------------  329
pattern 4  EVVKYFTDPNAVEPPDMELLYPFVDAALPLAYDMFLGRMINFQ-----------------------------------------------  313
pattern 5  EVVKYFTDPNAVEPPDMELLYPFVDAALPLAYGTCSLVE---------------------------------------------------  309
pattern 6  EVVKYFTDPNAVEPPDMELLYPFVDAALPLAYGVLGILLFHFQ----------RKLSIPYFIPNITLGSFGAITQFKSILPDRSTKVDIS  350
pattern 7  EVVKYFTDPNAVEPPDMELLYPFVDAALPLAYGVLGILLFH-------------------------------------------------  311
                            TM helix 2                        TM helix 3
                                                               intron 5       intron 6   NPDG motif
Wild-type  LAGPFAGAALSVSMFAVGLFLSTEPDAANDLVQVPSMLFQGSLLLGLISRATLGYAALHAATVSIHPLVIAGWCGLTTTAFNMLPVGCLD  450
pattern 1  ------------------------------------------------------------------------------------------
pattern 2  ------------------------------------------------------------------------------------------
pattern 3  ------------------------------------------------------------------------------------------
pattern 4  ------------------------------------------------------------------------------------------
pattern 5  ------------------------------------------------------------------------------------------
pattern 6  LAGPFAGAALSVSMFAVGLFLSTEPDAANDLVQVPSMLFQGSLLLGLISRATLGYAALHAATVSIHPLVIAGWCGLTTTAFNMLPVGCLD  440
pattern 7  ------------------------------------------------------------------------------------------
            TM helix 4                               TM helix 5                  TM helix 6
              intron 7                     intron 8          intron 9
Wild-type  GGRAVQGAFGKNALVTFGLSTYVMLGLRVLGGPLALPWGLYVLICQRTPEKPCLNDVTEVGTWRKALVGIALILVVLTLLPVWDELAEEV  540
pattern 1  ------------------------------------------------------------------------------------------
pattern 2  ------------------------------------------------------------------------------------------
pattern 3  ------------------------------------------------------------------------------------------
pattern 4  ------------------------------------------------------------------------------------------
pattern 5  ------------------------------------------------------------------------------------------
pattern 6  GGRAVQGAFGKNALVTFGLSTYVMLGLRVLGGPLALPWGLYVLICQRTPEKPCLNDVTEVGTWRKALVGIALILVVLTLLPVWDELAEEV  530
pattern 7  ------------------------------------------------------------------------------------------
                              TM helix 7                                  TM helix 8
                intron 10
Wild-type  GIGLVTTF   (548 aa)
pattern 1  --------   (326 aa)
pattern 2  --------   (342 aa)
pattern 3  --------   (329 aa)
pattern 4  --------   (313 aa)
pattern 5  --------   (309 aa)
pattern 6  GIGLVTTF   (538 aa)
pattern 7  --------   (311 aa)
```

Supplementary Figure S3. Alignment of predicted amino acid sequences of *A. thaliana* wild-type- and *egy1-4*-derived EGY1 protein. In *egy1-4*, *EGY1* encoded seven different polypeptides caused by impaired splicing events (pattern 1 to pattern 7). Sequence alignment was performed using ClustalW. Identical amino acids are marked by inverted letters using Boxshade (https://junli.netlify.app/apps/boxshade/), while unmatched amino acids are marked in red. Relative positions of introns (arrowheads), transmembrane (TM) helices (blue lines), and the conserved GNLR, HEXXH and NPDG motifs found in the membrane-bound metalloproteases, respectively (Chen et al., 2005; green lines), are also indicated.

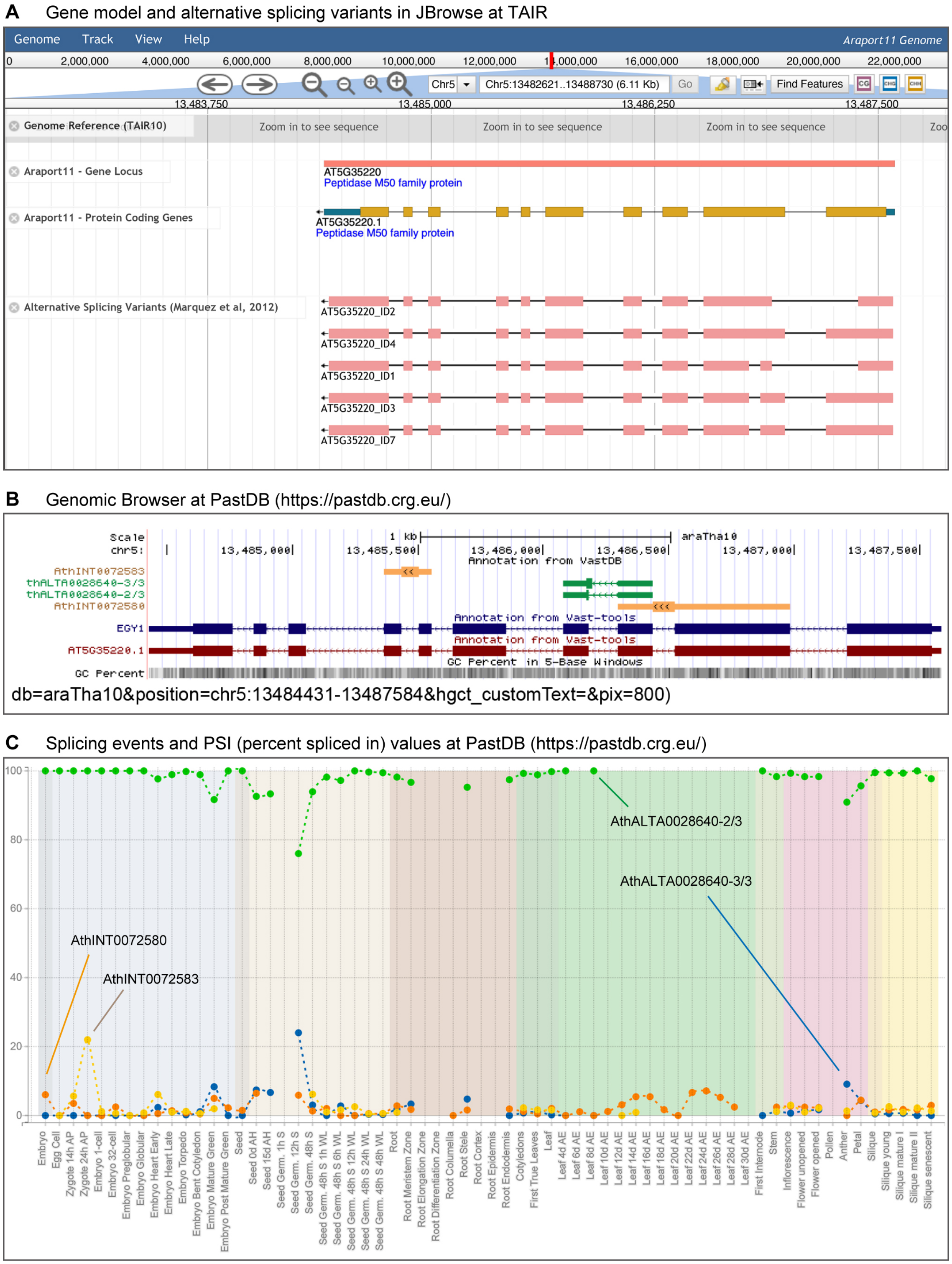

Supplementary Figure S4. The gene model of *EGY1* (At5g35220.1) and its splicing variants in public databases. (A) Presentation by the JBrowse platform at the TAIR site (https://www.arabidopsis.org, accessed in June 2024). (B) Presentation in the GENOMIC BROWSER at PastDB site (https://pastdb.crg.edu/, accessed in April 2024). (C) Presentation in the EVENT AND AS PROFILE at PastDB. In (A–C), images at each web site were saved and clipped. In (C), the four splicing events are denoted in the image for clarification. For details of the splice sites, see Supplementary Figures S2 and S3.

A AthALTA0028640-2/3

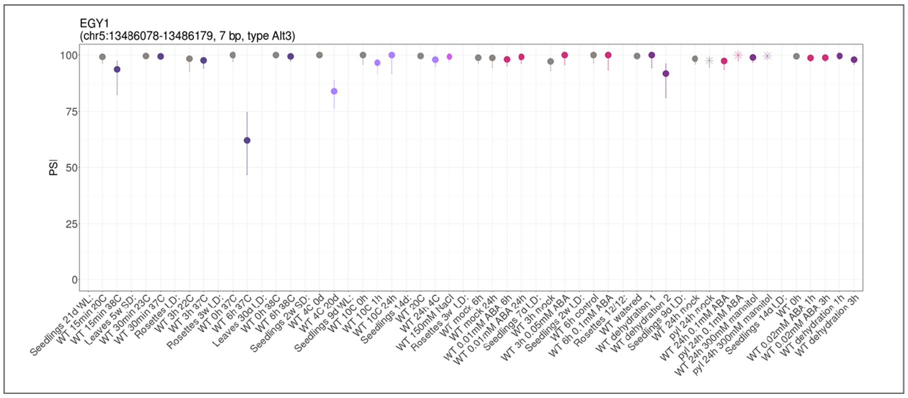

B AthALTA0028640-3/3

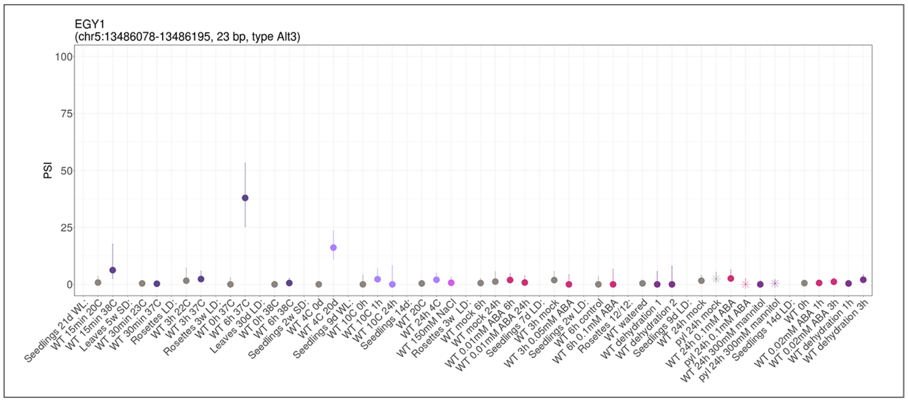

C AthINT0072580

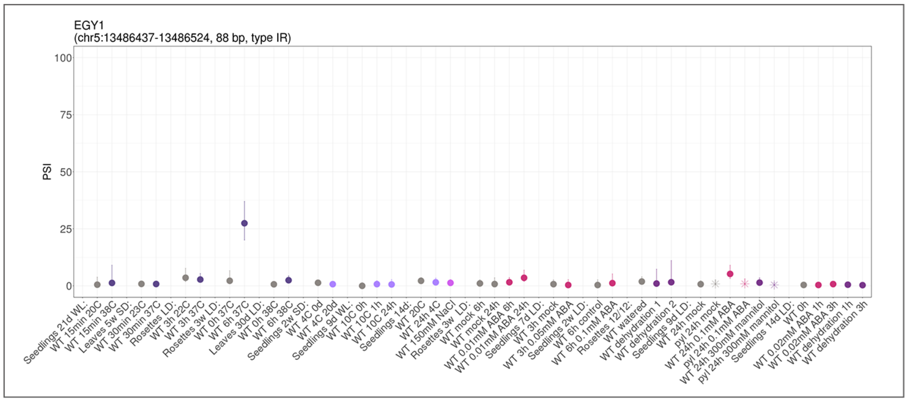

D AthINT0072583

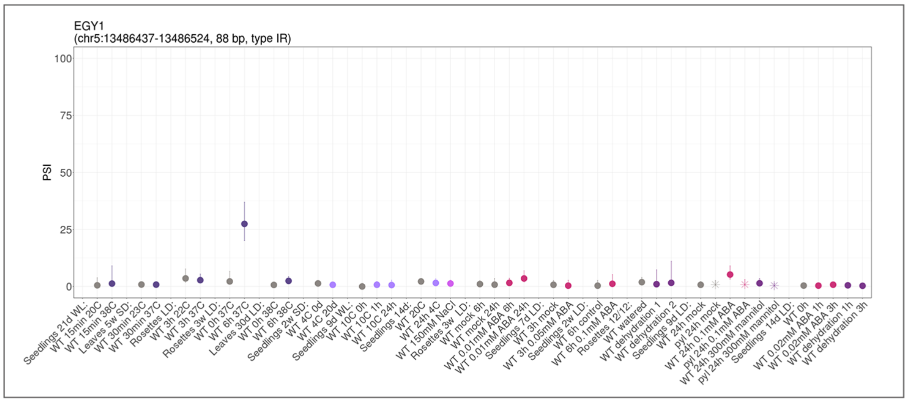

Supplementary Figure S5. PSI (percent spliced in) profiles for splice variants of EGY1 under abiotic stresses. (A) AthALTA0028640-2/3 (At5g35220.1). (B) AthALTA28640-3/3. (C) AthINT0072580. (D) AthINT0072583. These data were compiled by Martin et al. (2021) and obtained from the PastDB site (https://pastdb.crg.edu/, accessed in April 2024).

Supplementary Table S1. List of oligonucleotide primers used for RT-PCR analyses. Primers 1F to 9R were bound to *EGY1* (AT5G35220), while AtEF1a_F and AtEF1a_R were bound to *EF1A* (AT1G07940). Both 2nd-F and 2nd-R were used for next-generation sequencing. For these primers, 'F' represents a forward primer, while 'R' represents a reverse primer. For the information of recognition sites of *EGY1*-specific primers, see Supplementary Figure S1.

Primer name	Sequence (5' ➔ 3')
1F	CGCTCCCACATAAACGTTTCCC
2F	CTCCCTCGAGATGGGGACTCTCACCAGC
3F	GCCTTTAGCTTATGGTGTATTGG
4R	AGTTGCTCTGCTGATGAGTCC
5R	AGAGTTGCTCTGCTGATGAG
6F	GTCTGTTCCTATCTACAGAACC
7R	CCCTAACTACAACGTCTGCAC
8F	TACACGACGCTCTTCCGATCTGTAGATGCTGCATTGCCTTTAG (underline: recognition site of 2nd-F primer)
9R	AGACGTGTGCTCTTCCGATCTGTACTCCGGTCAGGAAGAATC (underline: recognition site of 2nd-R primer)
2nd-F	AATGATACGGCGACCACCGAGATCTACACxxxxxxxxACACTCTTTCCCT ACACGACGCTCTTCCGATCT (x: index sequence)
2nd-R	CAAGCAGAAGACGGCATACGAGATxxxxxxxxGTGACTGGAGTTCAGAC GTGTGCTCTTCCG (x: index sequence)
AtEF1a_F	AGGCTGGTATCTCTAAGGATGGTCA
AtEF1a_R	GGATTTTGTCAGGGTTGTATCCG

Supplementary Table S2. The genomic location, length, and splice site sequences of introns in the *Arabidopsis EGY1* gene [a].

Intron no.	Genomic region[b]	Length (bp)	5’ splice site		3’ splice site	
			Exon	Intron	Intron	Exon
1	337–566	230	ATG	GTACACA	TCCTCAG	CCA
2	1,023–1,10	88	CAG	GTACATG	TTAACAG	ATT
3	1,252–1,367	116	CAT	GTAAGTA	TTTGCAG	GAA
4	1,470–1,694	225	CAG	GTAATAA	TGTACAG	TTT
5	1,907–1,991	85	TGC	GTAAGCT	ATTGCAG	AGC
6	2,043–2,112	70	ATG	GTAGTGT	TTGTTAG	GTG
7	2,183–2,493	311	CAG	GTACTTC	TGTGCAG	GGA
8	2,563–2,649	87	GTG	GTATGTT	TTTTCAG	CTC
9	2,701–2,782	82	CAG	GTACACA	TGTACAG	AGA

[a] The gene model of *EGY1* consists of 10 exons and 9 introns (Chen et al., 2005; https://www.arabidopsis.org).

[b] The ‘A’ in the start codon of *EGY1* at exon 1 is defined as +1.

Supplementary Table S3. List of mutated genes in *egy1-4*. Mutations located outside the known coding regions of genes (e.g. promoter regions) are excluded. AGI codes and gene descriptions were compiled from the TAIR site (https://www.arabidopsis.org, accessed in November 2023).

No.	Zygosity	AGI code	Gene name	Description	Detection algorithm
**1**	Homo	AT5G43850	-	RmlC-like cupins superfamily protein	pindel & SNV-GATK
**2**	Hetero	AT3G61680	-	alpha/beta-Hydrolases superfamily protein	pindel & SNV-GATK
**3**	Hetero	AT4G17390	RPL15B	Ribosomal protein L23/L15e family protein	SNV-GATK
**4**	Hetero	AT4G14090	UGT75C1	UDP-Glycosyltransferase superfamily protein	SNV-GATK
**5**	Hetero	AT4G11720	HAP2	hapless 2	SNV-GATK
**6**	Hetero	AT3G15880	TPR4	WUS-interacting protein 2	SNV-GATK
**7**	Hetero	AT1G27110	-	Tetratricopeptide repeat (TPR)-like superfamily protein	SNV-GATK
